# Snowball ICA: A Model Order Free Independent Component Analysis Strategy for Functional Magnetic Resonance Imaging Data

**DOI:** 10.3389/fnins.2020.569657

**Published:** 2020-09-18

**Authors:** Guoqiang Hu, Abigail B. Waters, Serdar Aslan, Blaise Frederick, Fengyu Cong, Lisa D. Nickerson

**Affiliations:** ^1^School of Biomedical Engineering, Faculty of Electronic Information and Electrical Engineering, Dalian University of Technology, Dalian, China; ^2^Brain Imaging Center, Mclean Hospital, Belmont, MA, United States; ^3^Department of Psychiatry, Harvard Medical School, Boston, MA, United States; ^4^Department of Psychology, Suffolk University, Boston, MA, United States; ^5^School of Artificial Intelligence, Faculty of Electronic Information and Electrical Engineering, Dalian University of Technology, Dalian, China; ^6^Key Laboratory of Integrated Circuit and Biomedical Electronic System of Liaoning Province, Dalian University of Technology, Dalian, China; ^7^Faculty of Information Technology, University of Jyvaskyla, Jyvaskyla, Finland

**Keywords:** independent component analysis, functional magnetic resonance imaging, model order, dimension reduction, mutual information

## Abstract

In independent component analysis (ICA), the selection of model order (i.e., number of components to be extracted) has crucial effects on functional magnetic resonance imaging (fMRI) brain network analysis. Model order selection (MOS) algorithms have been used to determine the number of estimated components. However, simulations show that even when the model order equals the number of simulated signal sources, traditional ICA algorithms may misestimate the spatial maps of the signal sources. In principle, increasing model order will consider more potential information in the estimation, and should therefore produce more accurate results. However, this strategy may not work for fMRI because large-scale networks are widely spatially distributed and thus have increased mutual information with noise. As such, conventional ICA algorithms with high model orders may not extract these components at all. This conflict makes the selection of model order a problem. We present a new strategy for model order free ICA, called Snowball ICA, that obviates these issues. The algorithm collects all information for each network from fMRI data without the limitations of network scale. Using simulations and *in vivo* resting-state fMRI data, our results show that component estimation using Snowball ICA is more accurate than traditional ICA. The Snowball ICA software is available at https://github.com/GHu-DUT/Snowball-ICA.

## Introduction

Functional connectivity and network-based analysis of functional magnetic resonance imaging (fMRI) have revolutionized our understanding of the overall functional organization of the brain (Beaty et al., 2018; Pedersen et al., 2018; Sokolov et al., 2018). Independent component analysis (ICA), a commonly used data-driven approach for fMRI data analysis, has been effectively used for functional network studies (Seifritz et al., 2002; Hermans et al., 2011; Freeman et al., 2014; Richiardi et al., 2015; Constantinescu et al., 2016; Glasser et al., 2016; Rose et al., 2016; Tavor et al., 2016). However, there remains significant variation in spatial characteristics of networks between datasets and between ICA methods used to identify optimal configuration of components.

Model order selection (MOS – choosing the number of extracted components) in ICA is a significant methodological concern that contributes to this variation in fMRI brain network analysis (Abou-Elseoud et al., 2010; Beckmann, 2012; Kuang et al., 2018). Allen and colleagues (Allen et al., 2012) found that when the model order is too low, ICA distorts the estimation of sources. However, increasing the model order beyond the true dimensionality can result in certain sources being split into multiple components. In fact, most conventional signal processing applications used to estimate the number of source signals cannot reliably model the exact spatial characteristics of these signals, even when the true model order is known. This remains a significant problem for neuroscience researchers.

Many solutions have been proposed to address this issue. Information-theoretic criteria (ITC) have been used in numerous signal processing applications to estimate model order, including minimum code length based minimum description length (MDL) criterion (Rissanen, 1978), Akaike information criterion (AIC) (Akaike, 1998), and Bayesian information criterion (BIC) (Rissanen, 1978). Combining ITC with a resampling method was proposed for subsampling a set of effectively independent and identically distributed (i.i.d.) samples from dependent data samples (Li et al., 2007). The Laplace approximation (LAP) algorithm (Minka, 2000) was improved based on the empirical distribution function of the eigenvalues developed in random matrix theory (Beckmann and Smith, 2004). Entropy-rate-based order selection by finite memory length model (ER-FM) and entropy-rate-based order selection by AR model (ER-AR) are two likelihood estimators-based methods, which use all available samples instead of down sampling data (Fu et al., 2014). All of these methods attempt to accurately estimate the number of intrinsic source signals, which is then used as the optimal model order before ICA decomposition is performed.

In the field of fMRI data processing, the Group ICA of fMRI Toolbox(GIFT^1^; Calhoun et al., 2001a) and the Multivariate Exploratory Linear Optimized Decomposition into Independent Components (MELODIC^2^; Beckmann and Smith, 2004) are the two most popular software tools used for ICA. Both of these tools implement a principal component analysis (PCA)-based data reduction step prior to ICA. Moreover, for fMRI data, ICA can be performed on single-subject fMRI data, or on multi-subject data (by either stacking the fMRI data across subjects or by temporally concatenating data across subjects). For resting state fMRI, which is most commonly used for network analysis, data are temporally concatenated across subjects prior to the analysis. In GIFT, the default option for data reduction of temporally concatenated multi-subject data proceeds by first reducing data at the individual subject-level with PCA, then applying PCA to the group-level (concatenated) reduced fMRI data for further data reduction prior to ICA. As the number of subjects and the length of the time series increases, in order to reduce required memory, several dimension reduction methods have been proposed (Calhoun et al., 2015). For example, multi power iteration (MPOWIT) was designed to estimate a subspace larger than the desired one (Rachakonda et al., 2016). In contrast, the default in MELODIC utilizes an incremental approach called Incremental Group PCA (MIGP) (Smith et al., 2014) to perform data reduction (although this option can be turned off to implement a two-stage group PCA-based data reduction). Even though initial data reduction differs between these methods, the model order is selected from the group PCA, and therefore determined prior to the ICA decomposition. The order of steps in the data reduction process may contribute to premature removal of data as “noise,” when it in fact contributes meaningful information.

In this article, we propose a new ICA strategy that does not require MOS before ICA decomposition, the Snowball ICA. This approach differs from traditional ICA algorithms by iteratively collecting information about source signals from the fMRI data. First, we demonstrate how conventional MOS procedures contribute to inaccurate estimation of signal sources. Our Snowball ICA will then be compared with standard implementations using MELODIC and GIFT to determine the accuracy of spatial quality estimation using simulated data and *in vivo* fMRI data.

## Materials and Methods

### Conventional Spatial Group ICA

For conventional multi-subject fMRI data analysis, both noise free ICA (Calhoun et al., 2001b) and probabilistic ICA (Beckmann and Smith, 2004) are widely used. Both of these ICA models suffer the effect of MOS (Abou-Elseoud et al., 2010; Beckmann, 2012). For simplicity, the spatial group ICA algorithm (Calhoun et al., 2001a) implemented as in GIFT is used to show how MOS will impact the extracted components. First, data reduction is performed for each subject:

(1) $$X_{\text{s}}=V_{\text{s}}^{-1}\,Z_{\text{s}},$$

where *Z*_s_ ∈ *ℝ*^*T*×*M*^ represents the *s*-th subject’s fMRI data after preprocessing, *T* is the number of volumes, *M* is the number of voxels of each scan. $V_{\text{s}}^{-1}$ is the dimension reduction matrix, which is usually obtained from eigen decomposition of covariance matrix of *Z*_s_. At this stage, components that explain 90% variance are typically used to construct the reduced data. For group ICA, data from different subjects is temporally concatenated after the data reduction, as follows:

(2) $$D=\left[\begin{array}{c} X_{1}\\ \vdots \\ X_{S} \end{array}\right]=\left[\begin{array}{c} V_{1}^{-1}\,Z_{1}\\ \vdots \\ V_{S}^{-1}\,Z_{S} \end{array}\right].$$

Singular value decomposition of the aggregate data is as follows:

(3) D=G⁢∑U,

where *D* ∈ *ℝ*^N×M^ is the aggregate matrix of all subjects fMRI data. *G* is a unitary *N*×*N* dimension reduction matrix. ∑ is a diagonal *N*×*N* matrix. Each value δ_k_ on diagonal of ∑ is the square root of variance of each component. *U* is an *N*×*M* matrix consisting of *N* unit row-vectors. ∑*U* represents the group PCA components.

**FIGURE 1:**
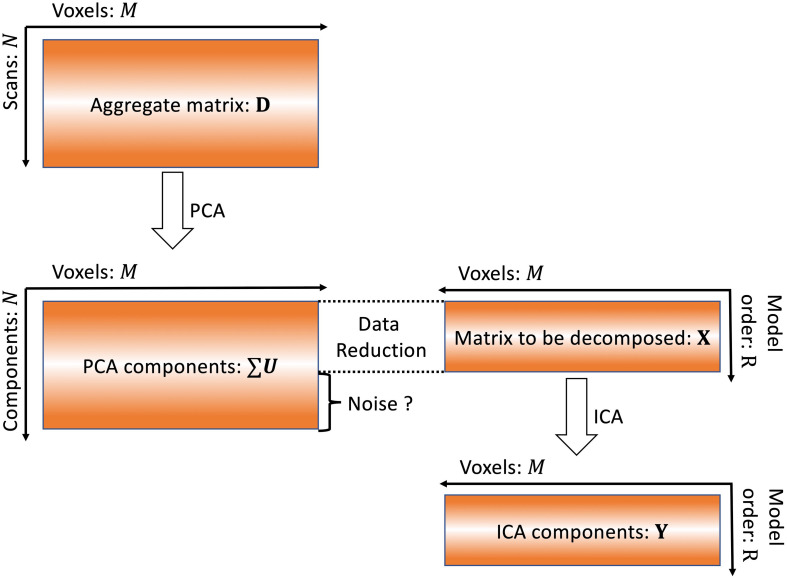
Conventional ICA algorithm. In conventional ICA, the model order is determined and then data reduction is applied prior to ICA. The removal of this information as “noise” may also result in removal of meaningful signals.

Based on PCA theory, the dimensionality of the aggregate matrix after concatenation is reduced again:

(4) $$X={\left(G_{:,1:R}\right)}^{T}\,D,$$

where R is the model order. The formula in Eq. 4 is therefore selecting the first R vectors of ∑*U*. The information contained in the *N-R* components is labeled as “noise” and removed as shown in Figure 1. In this study, we investigate whether or not these components are noise using simulated data.

Noise-free ICA is given by the following:

(5) Y=W⁢X,

where *X* ∈ *ℝ*^*R*×*M*^ is the matrix to be fed into ICA unmixing program. *W* ∈ *ℝ*^*R*×*R*^ represents the unmixing matrix. *Y* ∈ *ℝ*^*R*×*M*^ is the independent component matrix that is used to estimate the source matrix.

(6) $${\mathrm{GW}}^{-1}\,Y=\left[\begin{array}{c} V_{1}^{-1}\,Z_{1}\\ \vdots \\ V_{s}^{-1}\,Z_{s} \end{array}\right]=\left[\begin{array}{c} G_{1}\\ \vdots \\ G_{s} \end{array}\right]\,W^{-1}\,Y.$$

Reconstruction of time courses is done as follows:

(7) $$Z_{s}=V_{s}\,G_{s}\,W^{-1}\,Y.$$

With [*V*_*m*_*G*_*m*_*W*^−1^], (*m* = 1,2,⋯,*M*), containing the time course information of each component for each subject. *Y* represents the estimation of the source signals, including the spatial distribution of each component. The results from temporal concatenated Group ICA result in shared spatial distributions across subjects, with different temporal courses for each subject.

### Quantifying the Information Used in the ICA at a Given Model Order

Information included in the ICA algorithm is represented by the sum of the first *R* PCA components. When PCA is used as the data reduction procedure, the ratio of information used for ICA can be calculated with the following formula:

(8) $$\text{Ratio}\,\left(R\right)=\frac{\sum_{r=1}^{R}\delta_{r}}{\sum_{k=1}^{K}\delta_{k}},$$

where *R* is the model order and *K* is the dimensionality of the latent data contained in the algorithm. This index is used to describe the ratio of information included in the ICA procedure.

### Pseudo-ICA for Simulations

In order to study the distribution of the meaningful source information that may be contained in the PCA components labeled “noise,” a “pseudo-ICA” approach is used. In these simulations, the spatial correlation coefficient, *C*, between PCA components *U* and the ground truth signal sources is calculated, giving the following formula for pseudo-ICA:

(9) $$Y_{p}=C_{:,1:R}\,U_{1:R,:},$$

where *Y*_*p*_ represents the components matrix estimated by Pseudo-ICA. Each row of *Y*_**p**_ represents one component estimated by Pseudo-ICA. The order of the component matrix is exactly same as that of sources matrix. *R* represents the results calculated based on *R* PCA components.*U*_1:*R*,:_ is exactly the same with what would be fed into conventional ICA algorithms. *C*_*:,1:R*_ works as the unmixing matrix but without the limitation of independent constrain. Pseudo-ICA is therefore able to examine the effect of the number of PCA components on the accuracy of estimated results, neglecting the restriction of independence between components.

The correlation between the ground truth and PCA components represents the information ratio for each source signal for PCA components. Both the distribution of source information throughout the PCA components labeled “noise” and the accuracy of Pseudo-ICA components were used to evaluate performance of the PCA.

### Mutual Information Between Sources and Noise

Mutual information is a criterion used to describe the dependence between two variables. Under the independence constraint of spatial ICA, components with high mutual information will not be extracted appropriately. For two discrete random variables Y_1_ and Y_2_, the mutual information is defined as (Cover and Thomas, 2012):

(10) $$I\,\left(Y_{1},Y_{2}\right)=\sum_{y_{1}\in {𝒴}_{1}}\sum_{y_{2}\in {𝒴}_{2}}p_{\left(Y_{1},Y_{2}\right)}\,\left(y_{1},y_{2}\right)\,\log\left(\frac{p_{\left(Y_{1},Y_{2}\right)}\,\left(y_{1},y_{2}\right)}{p_{Y_{1}}\,\left(y_{1}\right)\,p_{Y_{2}}\,\left(y_{2}\right)}\right).$$

Once the model order was selected for the conventional ICA procedure, the PCA data reduction limits the resulting information for each source that is fed into ICA. For example, *R* PCA components will consist of information from *N* signal sources and *R-N* noise sources. However, the mutual information between different sources and the same noise will be different, with more spatially distributed sources having higher the mutual information with noise. This is demonstrated in section “Results” for the Simulations. As the number of PCA components increases, the number of noise signals increases, and the existence of spatially distributed sources sharing mutual information with noise also grows. In turn, because of the spatial independence constraint of ICA, large-scale sources may not be estimated. This effect is illustrated with simulations.

### Snowball ICA

**FIGURE 2:**
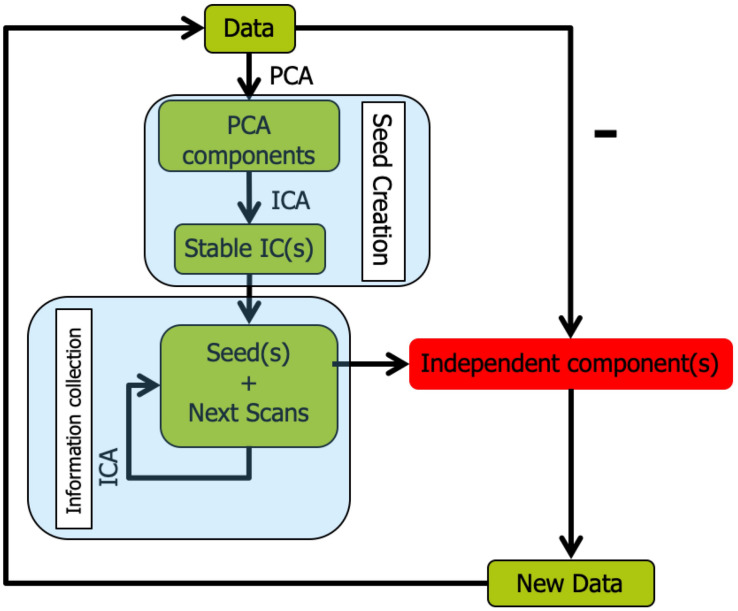
Snowball ICA algorithm. In order to avoid the necessity of specifying model order in conventional ICA, Snowball ICA estimates components one by one (or a few at a time) until no stable components can be further estimated from the data. After seed creation for a new component(s) to be estimated via information collection, the estimated snowball component(s) are removed from the multi-subject data and seed creation is repeated to determine the next seed pattern(s). The Snowball ICA will stop when there are no more stable components estimated during the seed creation step.

The overall workflow of Snowball ICA is illustrated in Figure 2. In Snowball ICA, the purpose is exactly the same as using conventional ICA to estimate independent component networks from fMRI data that are linear combination of network signals plus artifact (noise) signals. The aggregate fMRI data is denoted by *Z* ∈ *ℝ*^*T*×*M*^, which is organized with subjects’ fMRI data temporally concatenated without data reduction. *T* represents total number of scans across all subjects runs of fMRI data. *M* denotes the number of voxels. *T* is larger than the number of independent components *R* to be estimated, which means the model is overdetermined. However, all widely used ICA algorithms are determined (Bell and Sejnowski, 1995; Hyvarinen, 1999), made so by applying one of several dimension reduction methods. This data reduction step results in a loss of information. In contrast to conventional ICA, our proposed approach analyses aggregate fMRI data separated into different blocks to make the model determined. In order to combine blocks together, the Snowball ICA is divided into two parts. The first stage is seed creation, and the second stage is information collection. Seed creation is used to make sure the estimation is stable and information collection is then done to collect information from all blocks of data.

#### Seed Creation

The first stage is seed creation, in which a randomly selected individual subject’s fMRI data is selected from the group fMRI dataset. Since the information about each component from different subjects will be collected in the next stage (Information Collection), the initial seed creation is not that critical. It can be created only from a single subject, from large chunks of randomly selected data, or from conventional spatial group ICA results. In order to save time, we use a randomly selected subject to create the seed in this study. Once the data have been selected, ICA is repeated many times. The most stable component from these ICAs is selected as the seed. ICASSO (ICASSO-software package^3^) was used to evaluate component stability. The algorithm was run for ICA repeatedly with the same parameters and the same algorithm. Then hierarchical clustering was implemented to cluster all of the extracted components (Himberg et al., 2004; Zhang et al., 2018; Hu et al., 2019). Hierarchical clustering has been widely applied for the assessment of reproducibility of ICA components. The clustering method is the process for transforming a proximity matrix into a nested partition, which can be graphically represented by a tree called dendrogram. In this study, the dendrogram is formed from the bottom up. For this clustering method, at the first iteration, the number of clusters is same as the number of total independent components N. At the second iteration, the most similar clusters will merge as a new cluster, so the number of clusters will become N-1. At the third iteration, the number of clusters will become N-2. As the clustering goes forward, at the top level of the hierarchy, the number of clusters converges to a single cluster. Using this approach, the hierarchical organization of the data fed into the algorithm can be established. When hierarchical clustering is applied for ICA algorithm stability analysis, the cluster result is the dendrogram at the level of number of independent components. So, once the independent components from multiple runs are computed, no matter how many hierarchical clustering runs, the clustering results would be exactly same. After clustering, the difference between the average intra-class similarities and average inter-class similarities is used as an index to evaluate the stability of the components:

(11) $$I\,q\,\left(r\right)=\bar{S}\,{\left(r\right)}_{i\,n\,t}-\bar{S}\,{\left(r\right)}_{e\,x\,t}.$$

In order to ensure the stability of results, the most stable component was selected as the seed to feed into the information collection stage. *Y*_seed_ denotes the stable component estimated from Seed Creation. The Snowball ICA algorithm stops once stable components are no longer extracted.

#### Information Collection

In the second stage, information collection, the seed component is concatenated with randomly selected new scans, and these new aggregate data are then fed into the ICA unmixing algorithm. The resulting ICA component that most closely matches the seed is then used as the new seed to be concatenated with more of the original data in the next iteration. Gradually, the seed will collect all information about the signal it represents from each scan, resulting in accurate ICA components.

First, the aggregate data *Z* is separated into *K* blocks:

(12) $$Z=\left[\begin{array}{c} Z_{1}\\ \begin{array}{c} Z_{2}\\ \vdots \end{array}\\ Z_{K} \end{array}\right],$$

where *Z*_k_ ∈ *ℝ*^*T*/*K*×*M*^. Then for the first block, *Z*_*1*_, ICA with reference (ICA-R) (Lu and Rajapakse, 2005; Huang and Mi, 2007; Lin et al., 2007; Du and Fan, 2013) is implemented, with Y_seed_ being selected as the most stable component after repeated ICAs. Once the information belonging to the first block is collected, the estimated component is designated *Y*_*seed*_. Then the next block of data, *Z*_k_, goes through the same procedure to create an updated *Y*_seed_ new_ as reference for the next block, with remaining blocks going through the same procedure iteratively until all blocks are used. The order of processing blocks may be random. Once all blocks have been used, the resulting component is Y_1_, the first extracted ICA spatial map (SM). This process then repeats, after removing the estimated Y_1_ component from the original data, to identify the next component Y_2_, and so on. For each *Z*_k_, the implementation of ICA-R is as follow:

(13) $$\left[\begin{array}{c} Y_{s\,e\,e\,d\,\_\,n\,e\,w}\\ Y_{1}\\ \begin{array}{c} \vdots \\ Y_{T/K} \end{array} \end{array}\right]\leftarrow A_{k}\,\left[\begin{array}{c} Y_{\text{seed}}\\ Z_{k} \end{array}\right],$$

where *A*_k_ is unmixing matrix estimated with independence constraint. *Y*_*seed_ new*_ is updated seed that represents network information collected about *Y*_*seed*_ from *Z*_k_. It will replace *Y*_*seed*_ in the next block iteration. The seed component, *Y*_*seed*_, works as a reference and the information belonging to this reference network is gradually collected as more and more blocks are used. Once *K* blocks are used, the final *Y*_*seed*_ is the SM of estimated component of Snowball ICA and is represented by *S*_snowball_.

#### Removal of Estimated Components

As shown in Figure 2, once a component is accurately estimated, it is then removed from each subject’s fMRI data prior to determining the next seed to feed into Stage 2, as follows:

(14) $$Z_{s\,\_\,n\,e\,w}=Z_{s}-T_{s\,n\,o\,w\,b\,a\,l\,l}\times S_{s\,n\,o\,w\,b\,a\,l\,l},$$

where *S*_*snowball*_ is the accurate component estimated via the information collection stage of the Snowball ICA. There are several ways to reconstruct subject components from group ICA results (Erhardt et al., 2011; Du and Fan, 2013). Unlike the conventional ICA procedure, the unmixing matrix for each subject is difficult to obtain using Snowball ICA. However, Allen et al. (2012) showed that without any PCA dimension reduction prior to ICA (e.g., PCA is only used for rotation and whitening), the difference between back-projection and dual regression for reconstructing subject components is within computational precision. Hence, *T*_*snowball*_ represents time series that is calculated with the first stage of dual regression (Nickerson et al., 2017). *Z*_s_ represents the *s*-th subject’s fMRI data. For each subject, *Z*_*s_ new*_ replaces *Z*_s_in the next iteration of Snowball ICA to identify subsequent components. With this method, the information from components that has been estimated accurately will not be considered during creation of the next seed for new component estimation. Note that *Z*_*s_ new*_ is *only* used for seed creation, whereas the original data are used for information collection. This is done to account for the fact that different components may have some spatial overlap. In this case, the information from estimated components is only removed for seed creation, but not for information collection. Therefore, even though the overlapping signals are not included in the seed, the overlapping information will still be estimated during Information Collection.

The steps of Snowball ICA are as follows:

Step (1) Identify the seeds *Y*_*seed*_ as most stable ICA component (Iq > 0.9) from a small amount of the total data.
Step (2) Aggregate *Y*_seed_ with randomly selected scans chosen from all scans of all subjects to form a new data matrix and apply the ICA algorithm to decompose the new data matrix. Each scan is selected only once.
Step (3) The most similar IC(s) to the seed(s) are selected as new seed(s) *Y*_*seed*_.
Step (4) Repeat Steps 2 and 3 until all scans of all subjects have been used and the components are estimated accurately.
Step (5) Remove the resulting snowball ICA components (*S*_snowball_) from the multi-subject data and repeat to determine the next seed pattern. The Snowball ICA will stop when there are no more stable components estimated in Step 1.

### Evaluation of ICA Components

### Visualization

The ICA SMs are thresholded using a Z-threshold criterion (|*z*| > 2.3) (McKeown and Sejnowski, 1998; Calhoun et al., 2001a). These thresholded SMs are overlaid onto a transparent standard brain template to visualize the results. The MATLAB scripts used for this procedure can be downloaded from: https://github.com/GHu-DUT/Show_3D_GlassBrain.

#### Assessing the Snowball Component Spatial Patterns

For processing of real data that lack ground truth, the pros and cons of different algorithms can be compared as to whether they meet the ICA assumption. In theory, the purpose of ICA is to extract non-Gaussian signals. The stronger the non-Gaussianity of the signals, the more this assumption of ICA is satisfied. The standard measure of non-Gaussianity is kurtosis (Hyvärinen and Oja, 2000). The kurtosis of signal y with mean value μ and standard deviationσ is defined by:

(15) $$K\,u\,r\,t\,\left(y\right)=E\,\left[{\left(\frac{y-\mu }{\sigma }\right)}^{4}\right]=\frac{\mu_{4}}{\sigma_{4}}.$$

Independent component analysis spatial components are estimated by maximizing non-Gaussianity, therefore we use Kurt to evaluate the “goodness” of independence components. The higher the index is, the easier a network is distinguished from background (Gaussian) noise.

### Simulations

**FIGURE 3:**
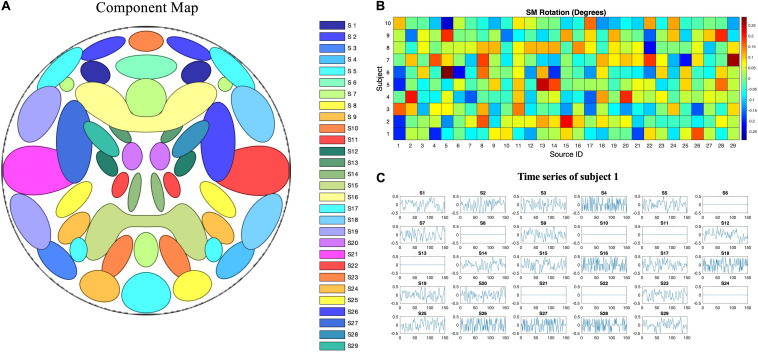
Ground truth spatiotemporal signal sources used in the simulations for simulated phantom fMRI data. **(A)** Spatial map showing the spatial configuration of the 29 signal sources. **(B)** To mimic between-subject spatial variability, the sources for each subject were given a small amount of random rotation and some sources were randomly excluded. **(C)** Temporal course of each signal source for a single subject. Straight lines with zero values indicate that the component is not present in that subject’s simulated data.

Simulated phantom fMRI data experiment aims to explore the reason that MOS effect the ICA decomposition. Simulated data were generated with the MATLAB toolbox, SimTB (Erhardt et al., 2012), which was developed to facilitate the testing of different analytic methods for multi-subject data and is freely available for download^4^. In SimTB, we adopt a data generation model that is largely consistent with the assumption of spatial ICA. In other words, data can be expressed as the product of activation temporal courses (TCs) and non-Gaussian sources, which we refer to as SMs. For subjects *i* = 1,⋯,*M*, we created *n* components, each consisting of an SM and corresponding TC. In our simulation, there are *M=10* subjects and *n=29* components. SMs have *V* = 148×148 voxels and TCs are *T=150* time points in length with a repetition time (TR) of 2s/sample. Rician noise with random contrast-to-noise ratio (CNR), selected according to a uniform distribution, for each subject was added for each time course (across subjects, mean ± SD: 0.32 ± 0.23). The simulated sources are shown in Figure 3. Voxels with values larger than 2.3 after standard normalization are defined as the signal represented by the component. The spatial extent of each network is defined as the number of voxels in the thresholded regions. Readers are pointed to (Erhardt et al., 2012) for more details of the spatial and temporal properties of the simulated sources.

In the traditional ICA algorithm, it is generally true that the first *R* PCA components will contain almost all of the information about all of the signal sources (the rest will correspond to noise/artifacts). PCA components are orthogonal, and the spatial cross correlation between PCA components and the ground truth signals can be used to identify the information about each signal contained in a PCA component.

For the simulated data, Matlab FastICA (Hyvärinen and Oja, 2000) was used as a representative traditional approach with model orders of 10, 29, 50, 100, 200, 400, 500, 800, and 1000, in order to compare the results under different model orders. The mutual information of sources with noise was also calculated. In Snowball ICA, for seed creation the model order was chosen as 10 and ICA was run 10 times with ICASSO. The stable components (Iq > 0.9) were used as seeds.

In order to test the performance of Snowball ICA, MELODIC, and GIFT under different CNR levels, a range of CNR (0.1–20) was also applied when subjects’ data generated. All the other parameters were kept exactly same. All three methods were then applied to the same dataset. In MELODIC and GIFT, the number of independent components was set to equal the number of sources (29 for the simulated data). Estimation accuracy is calculated as the average over all components of the spatial cross correlation between the independent components and their corresponding ground truth signals.

### Resting-State fMRI Data

Resting state fMRI for 50 healthy unrelated subjects were utilized from the WU-Minn Human Connectome Project (HCP: Van Essen et al., 2013) to demonstrate our new method. Each subject completed resting state fMRI with the following scan parameters: TE/TR/FA = 33.1 ms/720 ms/52°, 72 slices, 2 mm isotropic, eyes open fixation. The data was then temporally preprocessed and de-noised using the FIX approach (Griffanti et al., 2014; Salimi-Khorshidi et al., 2014). The resulting images were then aligned using MSM registration (Robinson et al., 2014). Full details of the HCP resting state data can be found in publications from the project (Smith et al., 2013; Van Essen et al., 2013).

Independent component analysis components estimated using the traditional ICA algorithm and the Snowball ICA strategy were compared. To apply the FastICA algorithm to the *in vivo* resting state fMRI data, FastICA as implemented in GIFT and in FSL MELODIC were both used with an empirical model order = 40. In Snowball ICA, FastICA was used for seed creation with a model order of 10 for Step 1. Stable components (Iq > 0.9) were used as seeds. In information collection, 20 scans were considered at the same time for Step 2. The estimated components from GIFT, MELODIC, and Snowball ICA were compared using visualization, kurtosis, and representativeness of network TCs.

To assess how selection of the initial parameters affects estimation, estimation consistency was tested across model orders in seed creation and across block sizes of information collection. For seed creation, model orders of 20, 40, and 60 were used to estimate seeds from randomly selected single subject’s fMRI data or from conventional group ICA SMs. The right frontoparietal network was then selected as the seed. For information collection, the block size was set to 20, 40, and 60. The consistency of the estimated networks with these parameter combinations was then assessed by visual inspection and similarity score, as shown in Figure 12. The similarity score is defined as the average Pearson correlation coefficient of SMs estimated with different parameters.

## Results

### Simulation Results

**FIGURE 4:**
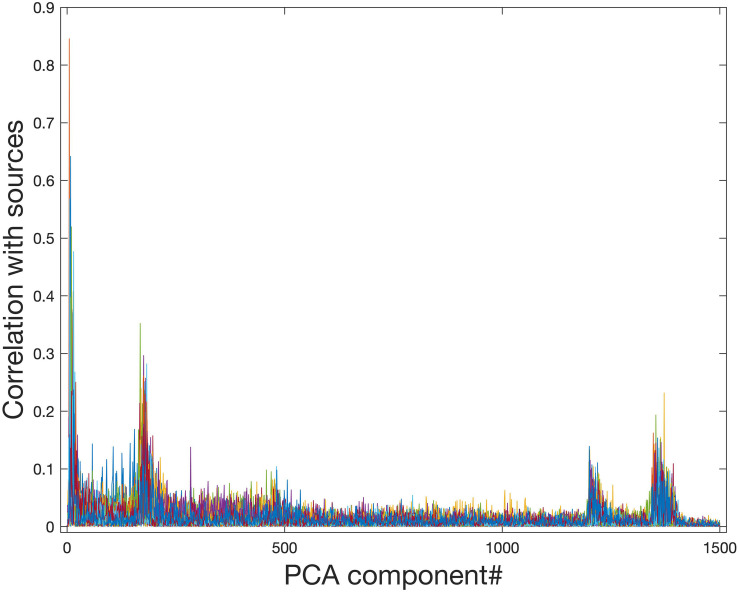
Correlation of PCA spatial maps with simulated signal source maps. Group PCA was applied to the simulated group data (*N* = 10) with 1500 components extracted in order of most variance accounted for. Even components extracted after approximately the first 200 and the first 1250 components still show a correlation with the simulated signal sources and therefore contain meaningful information about signal sources. When PCA is used for data reduction of group fMRI data, model orders may range from 20 to approximately 100–200, therefore this meaningful information will be lost during data reduction.

Ideally, the PCA data reduction procedure will be able to retain all information related to signal sources while removing only noise. Figure 4 shows that PCA data reduction is not effective for this purpose. Even for approximately the 200th and 1250th PCA components, they still contain meaningful information about signal sources. If the model order is chosen to be 29, which is exactly the number of sources in the simulated data, much of the information from signal sources is removed.

**FIGURE 5:**
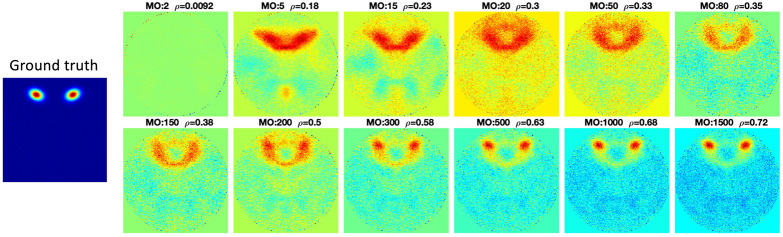
Pseudo-ICA results with different model orders. Without limitations of the spatial independence constraint of spatial ICA, the ICA estimation would be more accurate as more PCA components are retained with increasing model order (MO).

The second simulated signal source was selected as an example to show the Pseudo-ICA results under different model orders (Figure 5). Figure 5 shows that without the restriction of independence between components, when more PCA components are retained, the estimated component is more accurate.

**FIGURE 6:**
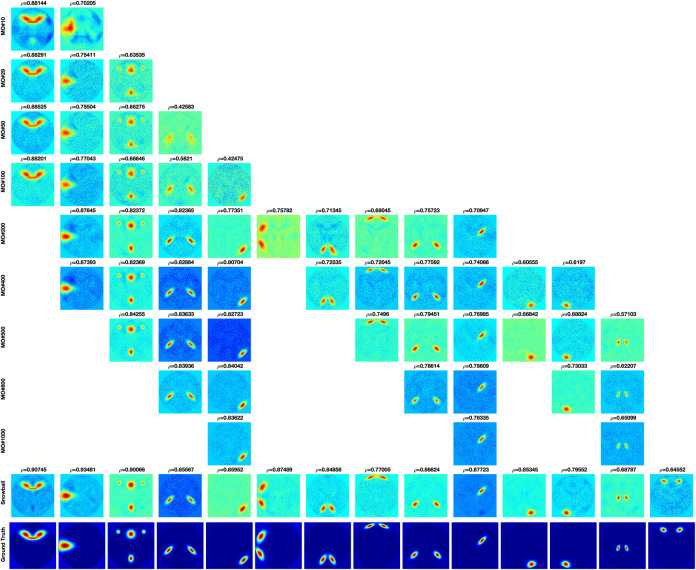
FastICA results under different model orders, MO (shown on the far left side of the figure). Snowball ICA results are shown in the row above the ground truth signals (shown in the bottom row). As model order increases for FastICA, the accuracy of estimation of individual networks increases but some networks with wide spatial distributions disappear at higher MO. In addition, FastICA only identifies a maximum of about 65% of the simulated sources, and only 9/29 at the true MO of the simulated data. In contrast, Snowball ICA estimates 26/29 ground truth signal sources, including widely distributed and highly focal networks, with high accuracy. Additional details on this figure can be found in the Supplementary Material.

The components estimated by Matlab FastICA under different model orders are shown in Figure 6. For each component, as model order increases, the accuracy of the estimated components also increases. However, when the model order is higher than 200, even though some new components are estimated, other components disappear. The disappearing components have relatively large scales.

**FIGURE 7:**
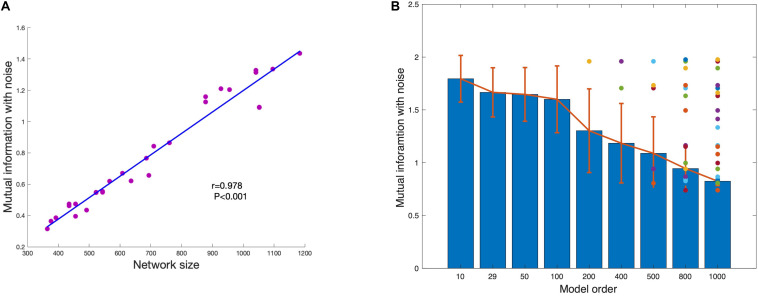
**(A)** The strong association between network size and shared mutual information with noise shows that more spatially extensive brain networks share greater mutual information with background noise. **(B)** The average mutual information of estimated networks under each model order. The colored dots represent the mutual information with noise of networks that disappear at higher MO with FastICA.

The disappeared component under each model order is defined as the component that cannot be estimated under the model order but can be estimated with lower model order. This means that the disappearance is caused by independent constraint but not information insufficient of the component. As shown in Figure 7A, the mutual information of network is significant correlated with network size. As the network size becomes larger, the mutual information between network and noise also increases. The average of mutual information with noise of estimated networks under different model order are shown in Figure 7B. The result shows that the mutual information with noise of estimated networks is decreasing as the model order increases. When the change of model order is relatively small, the mutual information with noise does not change much such as model order from 10 to 100. But when the change of model order value is relatively large, mutual information with noise also drops sharply, such as model order from 100 to 1000. The color dots represent the mutual information with noise of disappeared components at each model order. Almost all the disappeared components have higher mutual information with noise. We draw the conclusion that the scale of the estimated components is different under different model order. Limited with mutual information with noise, the higher the model order, the smaller the scale.

The comparison of Snowball ICA and traditional FastICA results are shown in Figure 6. For Snowball ICA, almost every component shows accurate estimation of sources with high correlation with ground truth. In order to compare the performance of Snowball ICA and conventional ICA, the number of extracted components that significant correlated ground truth was counted, with the threshold of 0.4 of Pearson correlation coefficient. In order to make sure the source was not split as several components, the components with the highest similarity correlated with ground truth are identified by eyes. Compared with the traditional ICA algorithm, Snowball ICA is able to estimate almost all signal sources (26/29) with high spatial accuracy. The signal sources include both large-scale networks (e.g., source #15, #16, #18, #21, #22) and small-scale networks (e.g., source #11, #12, #13, #28) can be estimated with Snowball ICA. For conventional ICA, a model order of 400, results in the most components being extracted (20/29). But this is still less than Snowball, and the accuracy is also lower. Meanwhile, 400 is much higher than the actual number of ground truth components. However, when the model order = 29, which is exactly same with the number of ground truth, the number of components obtained with conventional ICA are still few and the accuracy is also no better than the components that estimated with higher model order. From the results of Pseudo-ICA, for each source, when the PCA component contain more information of the source signals, the accuracy of estimated components shows significant improvement. For traditional ICA, limited by the restriction of independence for extracted components and influence of noise, the accuracy of its results is worse than Pseudo-ICA results. The higher the model order, the more pronounced the effect.

**FIGURE 8:**
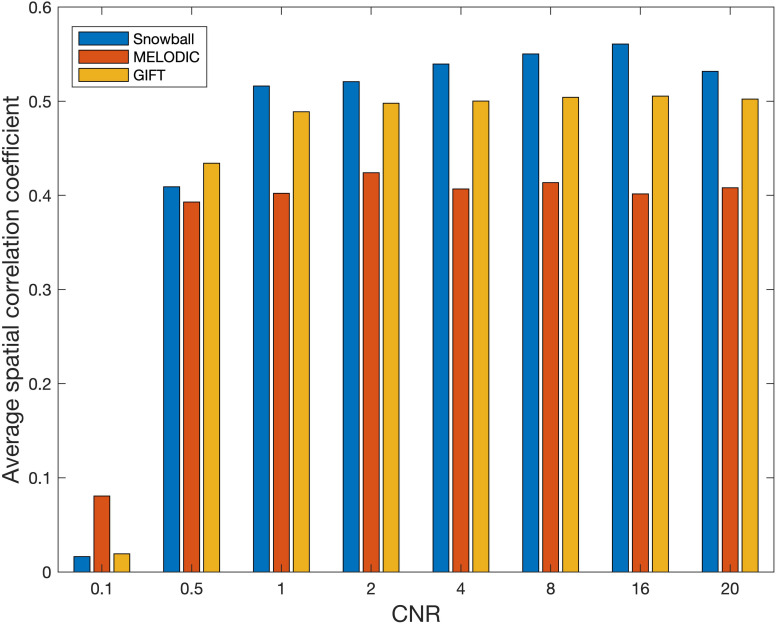
Spatial estimation accuracy of Snowball, MELODIC, and GIFT across different levels of contrast-to-noise ratio (CNR). Snowball ICA demonstrates greater accuracy across the ranges of CNR that may be observed in BOLD fMRI signals as GIFT and MELODIC.

The performance of Snowball ICA, MELODIC, and GIFT under different CNR levels are compared in terms of spatial estimation accuracy, as shown in Figure 8. Snowball ICA demonstrates greater accuracy across the ranges of CNR that may be observed in BOLD fMRI signals (Welvaert and Rosseel, 2013) as GIFT and MELODIC.

### Resting State fMRI Data Results

**FIGURE 9:**
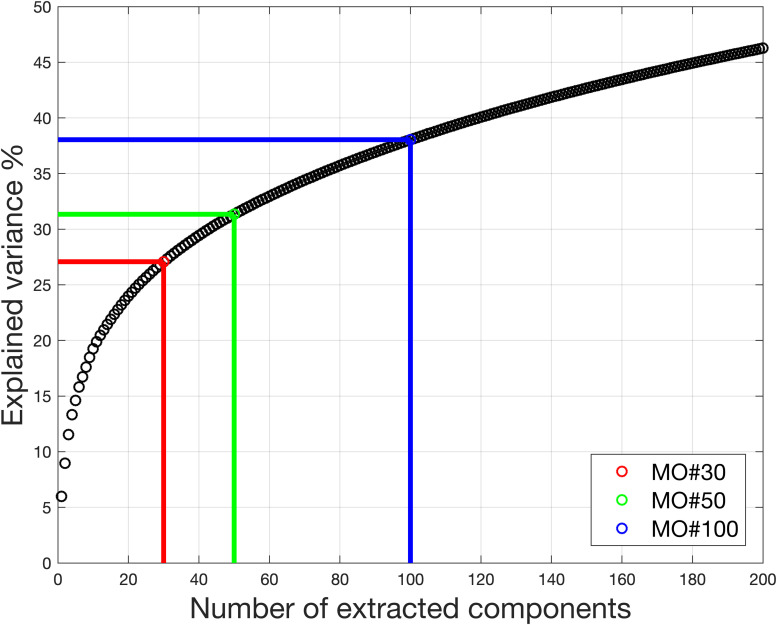
Real *in vivo* fMRI data result: variance retained under different model order. With model order equal to 30, only 27% of the variance is retained for the ICA. Even with model order equal to 100, the variance retained for the ICA is no more than 40%.

The model order was chosen from 25 to 50 for fMRI data processing (Beckmann et al., 2005; Damoiseaux et al., 2006; Luca et al., 2010). Figure 9 shows the information used under different model orders. Even when model order is 100, the information used is no more than 40%. That means more than 60% of the information was removed, reducing potential accuracy.

For the dataset, the estimated model order is found to be 16 using the MDL criteria, 648 using the LAP, 830 using ER_AR and 806 using ER_FM. The wide range of estimated model order when using different criteria makes MOS a big problem in real-world applications. The explained information under model order 16 is only 22.36%. Under the model order of 648, 830, or 806, based on simulation results, some components with large scale could not be extracted. Even though model order 830 is very high, the information used is only 71.34%.

**FIGURE 10:**
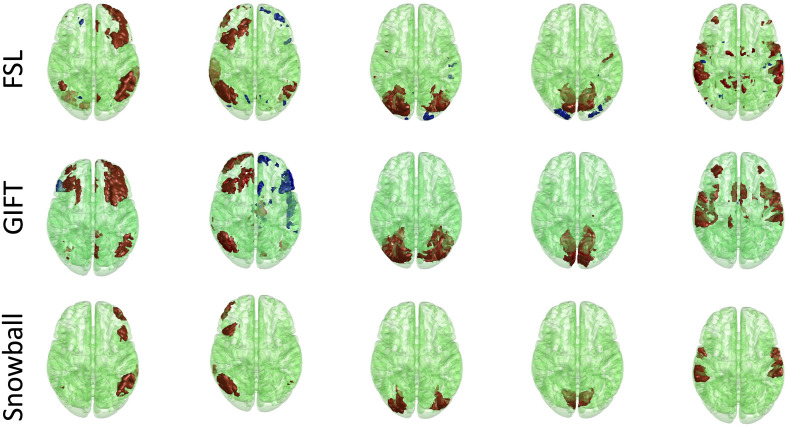
Real *in vivo* fMRI data results: assessing the Snowball component via visualization. Spatial maps of five networks extracted by FSL, GIFT, and Snowball. The threshold is 2.3 for all spatial maps, after standardization of spatial maps. Spatial maps of all components estimated from each method are shown in the Supplementary Material.

**FIGURE 11:**
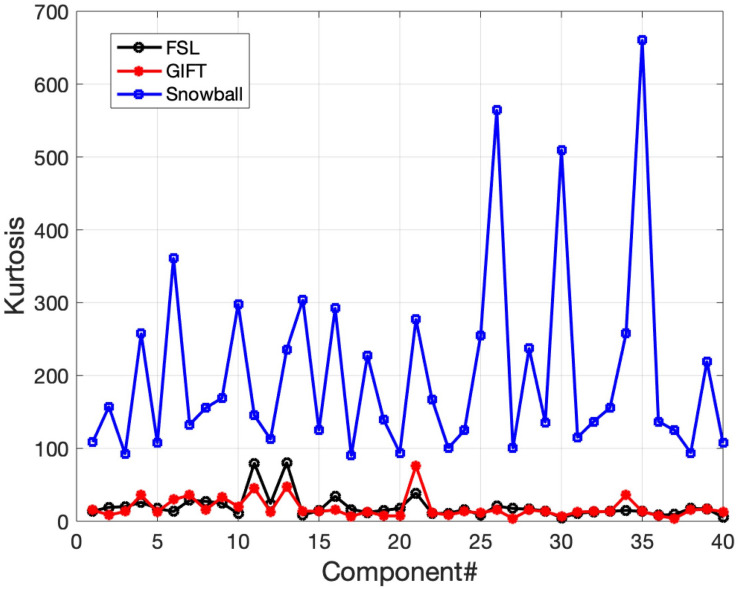
Real *in vivo* fMRI data results: assessing the Snowball component via kurtosis. Kurtosis for the set of spatial maps extracted by FSL, GIFT, and Snowball ICA. The results of Snowball ICA have the highest kurtosis and are thus most consistent with the assumptions of the ICA model.

The corresponding components estimated by Snowball ICA, GIFT, and MELODIC were compared by visual inspection and kurtosis. Visual inspection of components estimated by each method suggests that Snowball ICA produces SMs with the cleanest spatial distribution (Figure 10). Figure 11 shows that components estimated by Snowball ICA exhibit the greatest kurtosis, e.g., non-Gaussianity. However, Snowball ICA is a time-consuming strategy. MELODIC, GIFT, and Snowball were run on a computing cluster with 5 Linux-X64 nodes. The configuration of each node is: Intel(R) Xeon(R) Gold CPU 6130 2.10 GHz and 187.5 GB of random-access memory (RAM). The time costs of each method to estimate the same number of components are as follows: GIFT: 17 min, MELODIC: 50 min, Snowball: 30 h and 11 min. Further optimization of Snowball will be needed to reduce the computing time, however computing clusters are becoming more widely available and balance the need for improved methods for network estimation with intensive computational needs for our method.

**FIGURE 12:**
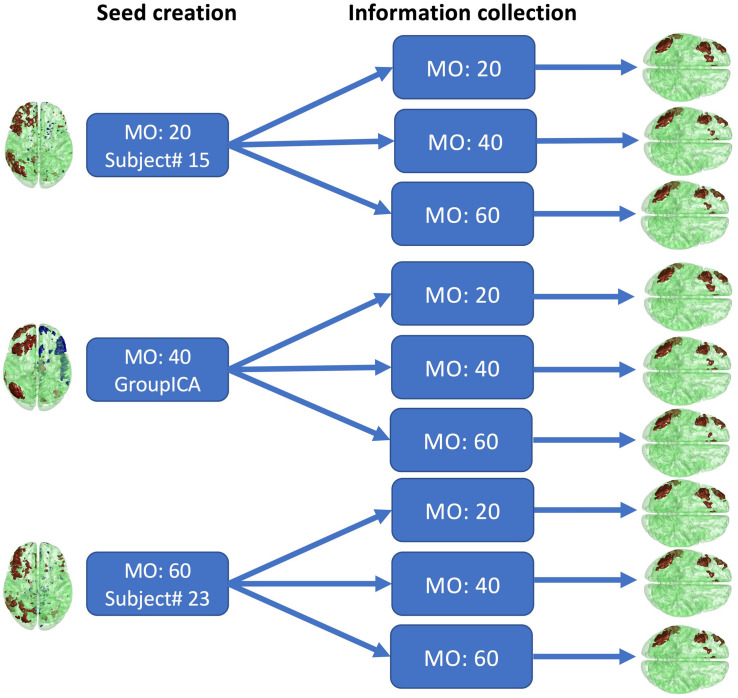
Real *in vivo* fMRI data results: Snowball ICA parameter selection. Comparison of the estimated right frontoparietal network obtained using different parameters for seed creation and information collection shows that although considerable differences exist between seeds estimated with different model orders in seed creation and in block sizes, the final estimated networks are highly similar.

The results of different parameter choices are shown in Figure 12. The seeds estimated with different model orders in seed creation from different subjects vary greatly, with an average similarity across three different seeds equal to 0.25. However, the final estimates of the right frontoparietal network after information collection are all highly similar, with an average similarity equal to 0.82, even when different block sizes are applied. As shown in Figure 12, after thresholding, the visualized results are nearly identical. Our findings demonstrate that different seeds constructed with different parameters in the seed creation step, and information collection with different parameters, will still converge to the same solution for a given network. As such, initial parameter selection is not a crucial step in implementing Snowball ICA.

## Discussion

The main findings of this study are that (1) traditional ICA methods have significant limitations in estimation of signal source number and spatial component quality, (2) information is lost during data reduction, and (3) large and small scale components cannot be accurately estimated with the same model order due to independence constraints. Furthermore, our proposed method, the Snowball ICA, addresses these limitations and outperforms traditional ICA algorithms on a number of metrics. We believe these findings are important for a number of reasons.

Information is lost by data reduction. When the model order is different, different numbers of PCA components are used, and the information fed into ICA unmixing program is markedly different. Therefore, the accuracy of components varies based on model order. Traditional ICA presumes that PCA data reduction reduces redundant information and only leaves useful information. This is easy to implement when the signal-to-noise ratio is high, such as mixed high-quality acoustic signals. However, because fMRI data is much more complex than acoustic signals, the removal of this information as “noise” may be obscuring meaningful signals in the brain and contributing to issues with reproducibility. While increasing the number of PCA components used increases the accuracy of components and the percentage of information used, traditional methods are unable to include all of the meaningful information. Snowball ICA is able to estimate these components accurately, represent higher proportions of information, and do so without increasing model order to unreasonable levels. Even though PCA is also applied in Snowball ICA seed creation part, the lost information would be collected in the information collection stage.

The spatial scale of estimated components decreases as the model order increases with conventional approaches. Based on information theory with independence constraints, the mutual information between components should be low (Hyvarinen, 1999; Hyvärinen and Oja, 2000). Each signal has intrinsic mutual information with noise. Larger-scale network has higher mutual information with noise. However, inevitably, once model order is selected both source information and noise components will also be extracted. Limited by mutual information with those noise, some components with large shape may not be extracted. From the simulations, when the model order is smaller, components with a larger scale can be extracted, but this is less accurate due to data reduction limitations. In contrast, Snowball ICA is able to extract both large- and small-scale components. This is especially important given the variation in network composition in the brain. Snowball ICA is not limited to research questions involving similarly scaled network interactions – it can be used to simultaneously study the large- and small-scale networks that coexist in the brain.

The information collection step of Snowball can be followed with any kind of current ICA. There are two kinds of ICA algorithms in fMRI data processing. The first is GIFT, based on the ICASSO, the conventional method introduced in this article. The other is probabilistic ICA (Beckmann and Smith, 2004), also known as noise ICA, which attempts to generalize ICA to include noise. The mechanism of model order effect of noise free ICA is explored in study. Probabilistic ICA leverages some information from noise, which may improve the accuracy of estimation to some extent. Further work is needed to investigate the how model order affects probabilistic ICA. Both of these two algorithms could be followed by Snowball ICA information collection, to improve the accuracy of the components and strengthen confidence of fMRI findings.

In addition to the application of ICA for fMRI data analyses, ICA is also widely used in the analysis and processing of electroencephalogram (EEG) and EEG-fMRI data fusion (Calhoun et al., 2009; Lei et al., 2012; Dong et al., 2014, 2016). For data fusion, spatial ICA of fMRI and temporal ICA of EEG has been used to extract features that are matched across the modalities. The number of independent components impacts the final estimations for both spatial ICA and temporal ICA. With Snowball ICA, the intrinsic embedded components can be estimated more accurately without the need to specify the model order for either EEG or fMRI, which may lead to improvements in fusion of high spatial and temporal resolution information.

When Snowball ICA is applied for group wise fMRI decomposition, there are several factors that may influence the accuracy of estimation. First, differences in brain shapes of different subjects may result in misalignment of the network SMs across subjects. Second, it is logical to assume some components are stronger in some subjects/scans. So, if iteration in information collection is not stable, the order where these scans are fed into Snowball may impact the estimation of components. Third, there are a number of parameters that are important for snowball ICA (seed model order, threshold Iq, block size of information collection iterations). In this study, theses parameters are selected based on our experience. The threshold Iq was also selected as 0.9 based on our experience and on previous published studies. Abou-Elseoud et al. (2010) who found that when the value of Iq is greater than 0.8, the results are repeatable. A study by Allen et al. (2011) also demonstrated that the Iq of meaningful components was typically larger than 0.9. In the present study, although the choice of specific Iq may affect the number of extracted components, use of a higher Iq ensures that the extracted components are reliable. In addition, the specific choice of Iq does not affect the accuracy of the estimated components. We also observed that the slight change of the model order will not have much influence on the estimated components (Figure 7B), which is also demonstrated with parameter selection (Figure 12). Hence, the selection of the seed model order and the block size of information collection iterations is not fatal.

Model order selection is also a crucial step in temporal ICA. Theoretically, Snowball ICA can also be applied for temporal ICA. However, it is not clear if the improvements we observe with spatial ICA will be realized with temporal ICA. In the case of spatial ICA, Snowball is effective at estimating networks without specifying a model order because it utilizes more information in the data (e.g., it obviates loss of information during data reduction with PCA) and is better able to identify networks that share large degree of mutual information with the noise. It is likely that temporal ICA will have different factors that may impact whether improvements in estimation would be seen with Snowball ICA.

While Snowball ICA is time-consuming due to the iterative process, it will be important for researchers determine the cost-benefit based on their hypothesized source signal scale. Further research will be necessary to explore ways to decrease computational costs for Snowball ICA. Besides, Snowball ICA is an empirical strategy that combining the conventional ICA and iteration of ICA with reference to solve the mode order problem. New algorithm with overall theoretical principles to solve the model order problem worth further investigation. Due to the lack of acknowledge of ground truth in real-world application, even though Snowball ICA can be identified with better performance compared with conventional ICA in some sense, more comparison of them in terms of neuroscience such as assessing relative heritability, or behavioral prediction accuracy, or split-half reproducibility is needed.

## Conclusion

In this article, we present a novel strategy, called Snowball ICA, to solve the MOS problem of ICA for applications to fMRI data processing. Choice of model order for ICA, and the PCA data reduction step prior to the ICA, directly impacts how much variance in the data is utilized for ICA estimation. In addition, shared mutual information between estimated sources and noise varies with the network spatial scale, making optimization of model order a challenging problem. Snowball ICA ultimately utilizes much more information contained in the data for the ICA decomposition, and is able to estimate signal sources that share mutual information with noise, which results in improved network estimation when compared with traditional ICA. The effectiveness of the proposed method is demonstrated through extensive simulations and by application to *in vivo* fMRI data.

## Data Availability Statement

Publicly available datasets were analyzed in this study. This data can be found here: https://www.humanconnectome.org.

## Author Contributions

GH, FC, and LN: conceptualization. GH: methodology, software, formal analysis, and writing—original draft preparation. GH, SA, and BF: validation. GH and LN: investigation. GH, AW, and LN: writing—review and editing. AW: visualization. FC and LN: supervision and project administration. FC, LN, and BF: funding acquisition. All authors contributed to the article and approved the submitted version.

## Conflict of Interest

The authors declare that the research was conducted in the absence of any commercial or financial relationships that could be construed as a potential conflict of interest.

## Footnotes

1 https://trendscenter.org/software/gift/

2 http://fsl.fmrib.ox.ac.uk/fsl/fslwiki/MELODIC/

3 http://www.cis.hut.fi/projects/ica/icasso/

4 https://trendscenter.org/software/
