# Supplementary material for: Snowball ICA: A Model Order Free Independent Component Analysis Strategy for Functional Magnetic Resonance Imaging Data
Source: Front Neurosci. 2020 Sep 18;14:569657. doi: 10.3389/fnins.2020.569657 (PMC7530342; doi:10.3389/fnins.2020.569657)
Supplement: Supplementary file 1 [file Data_Sheet_1.PDF]

Snowball ICA estimates 80 components (without specifying a priori a model order). We show the full set of Snowball ICA spatial maps below. Consistent with published literature implementing group ICA of fMRI data using either MELODIC or GIFT that specifies model order of approximately 20-40 to identify large-scale brain networks, we show GIFT and MELODIC spatial maps at a model order of 40. For completeness, we also show GIFT and MELODIC maps with a model order matched to the number of components estimated by Snowball ICA, e.g., 80 (which is not a typical model order for GIFT and MELODIC as reported in the literature). Spatial maps estimated by Snowball are less "noisy", and are more spatially localized than maps estimated by GIFT and MELODIC at either dimensionality. All spatial maps are shown at the same threshold, Z = 2.3.

Independent components with Snowball ICA:

The following are 80 spatial maps of independent components estimated with Snowball ICA.

#1 #2 #3 #4

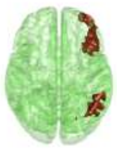
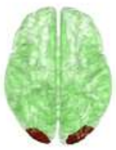
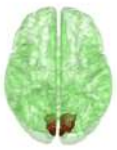
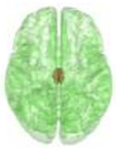

#5 #6 #7 #8

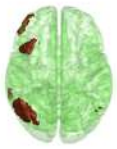
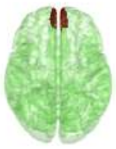
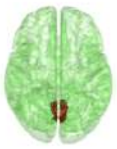
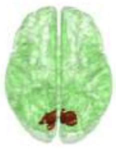

#9 #10 #11 #12

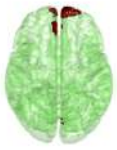
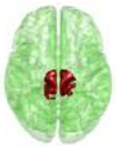
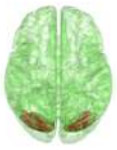
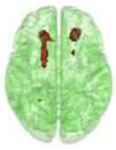

#13 #14 #15 #16

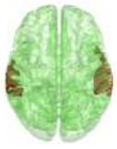
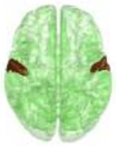
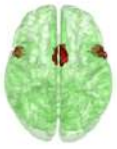
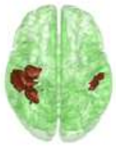

#17

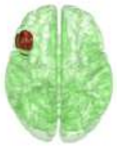

#18

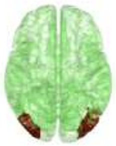

#19

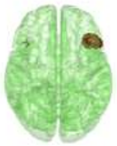

#20

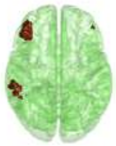

#21

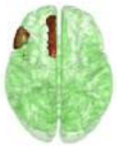

#22

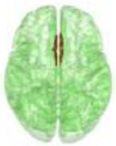

#23

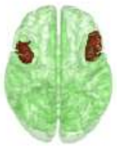

#24

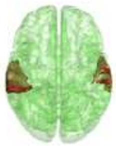

#25

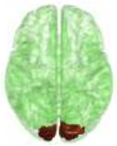

#26

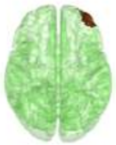

#27

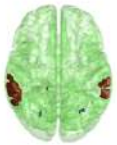

#28

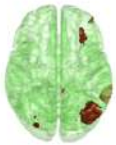

#29

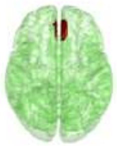

#30

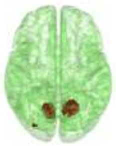

#31

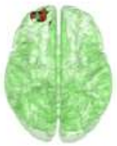

#32

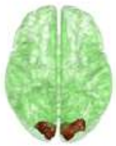

#33

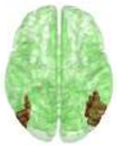

#34

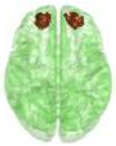

#35

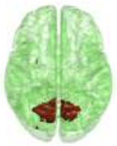

#36

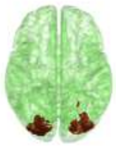

#37

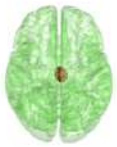

#38

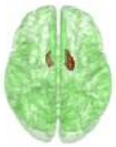

#39

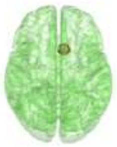

#40

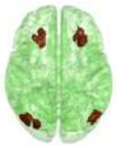

#41

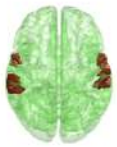

#42

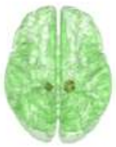

#43

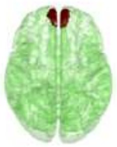

#44

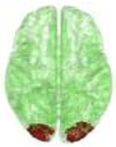

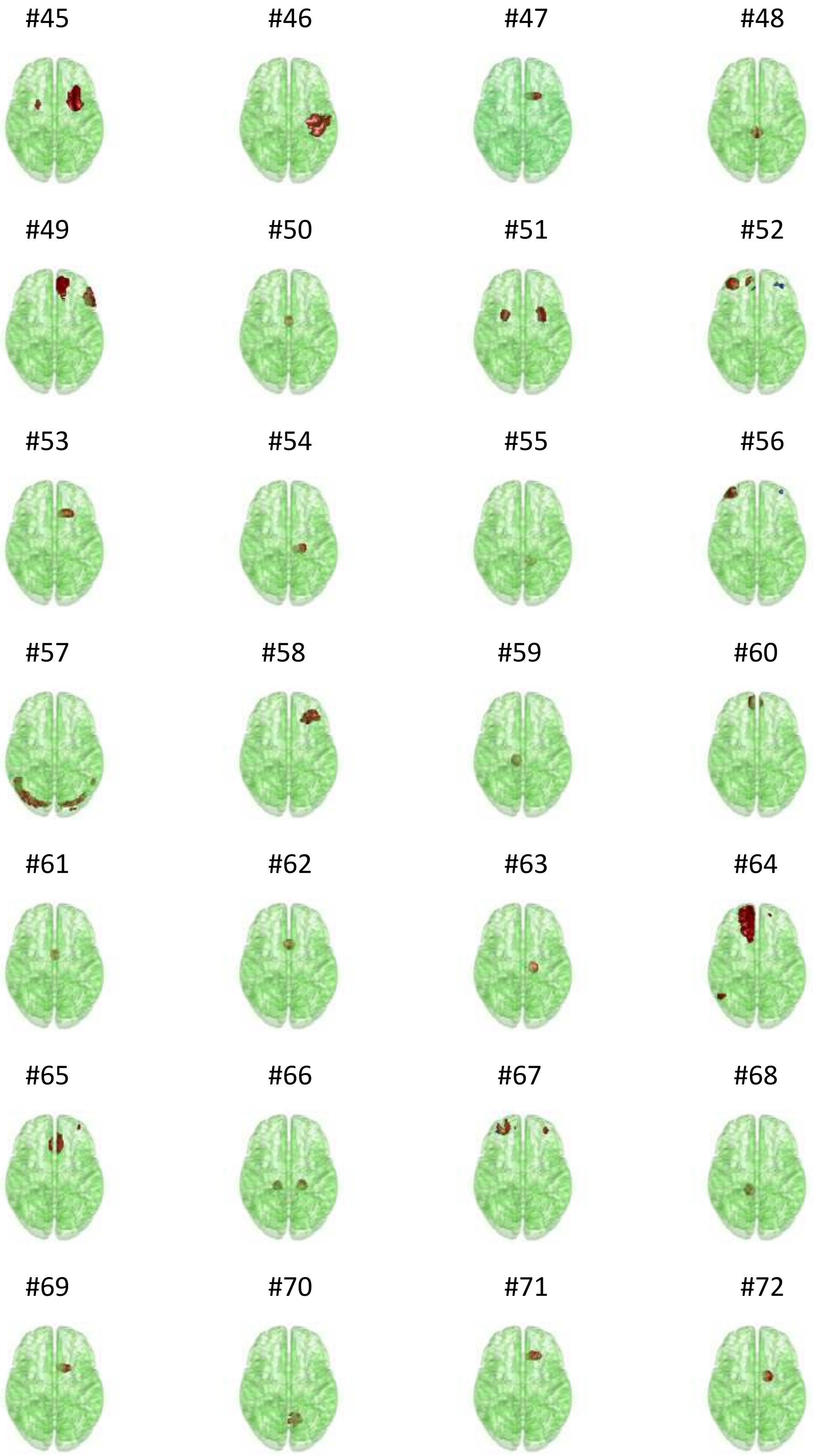
#45
#46
#47
#48
#49
#50
#51
#52
#53
#54
#55
#56
#57
#58
#59
#60
#61
#62
#63
#64
#65
#66
#67
#68
#69
#70
#71
#72

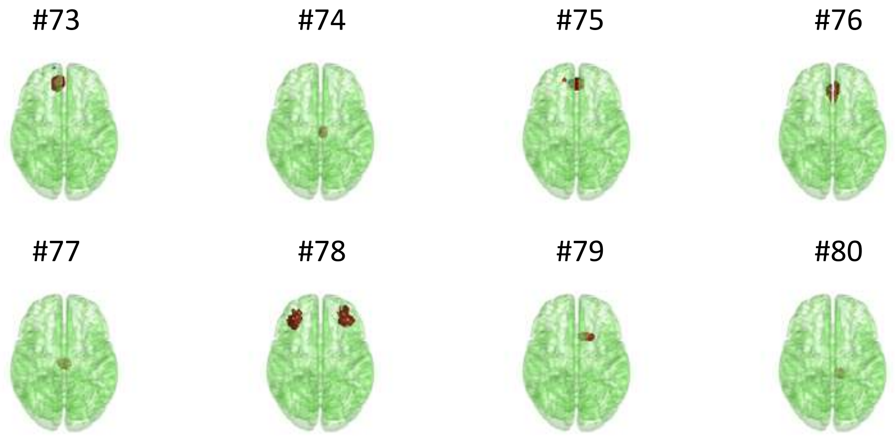

Independent components with GIFT:

The following are spatial maps of independent components estimated with GIFT when model order is selected as 40.

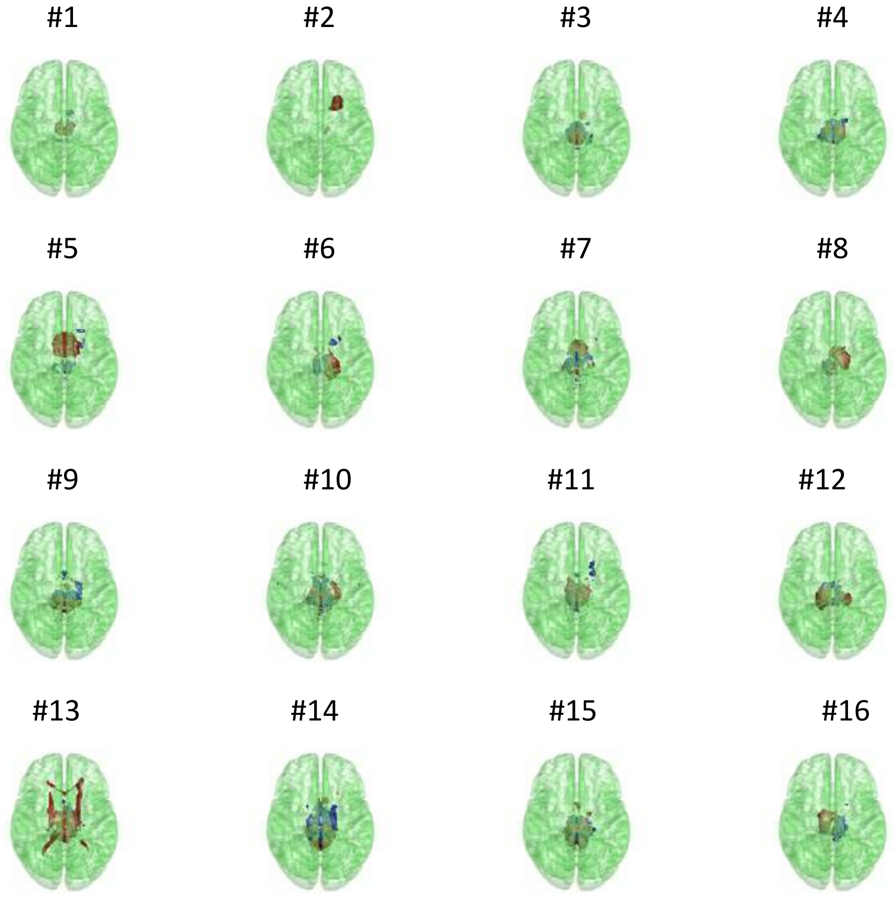

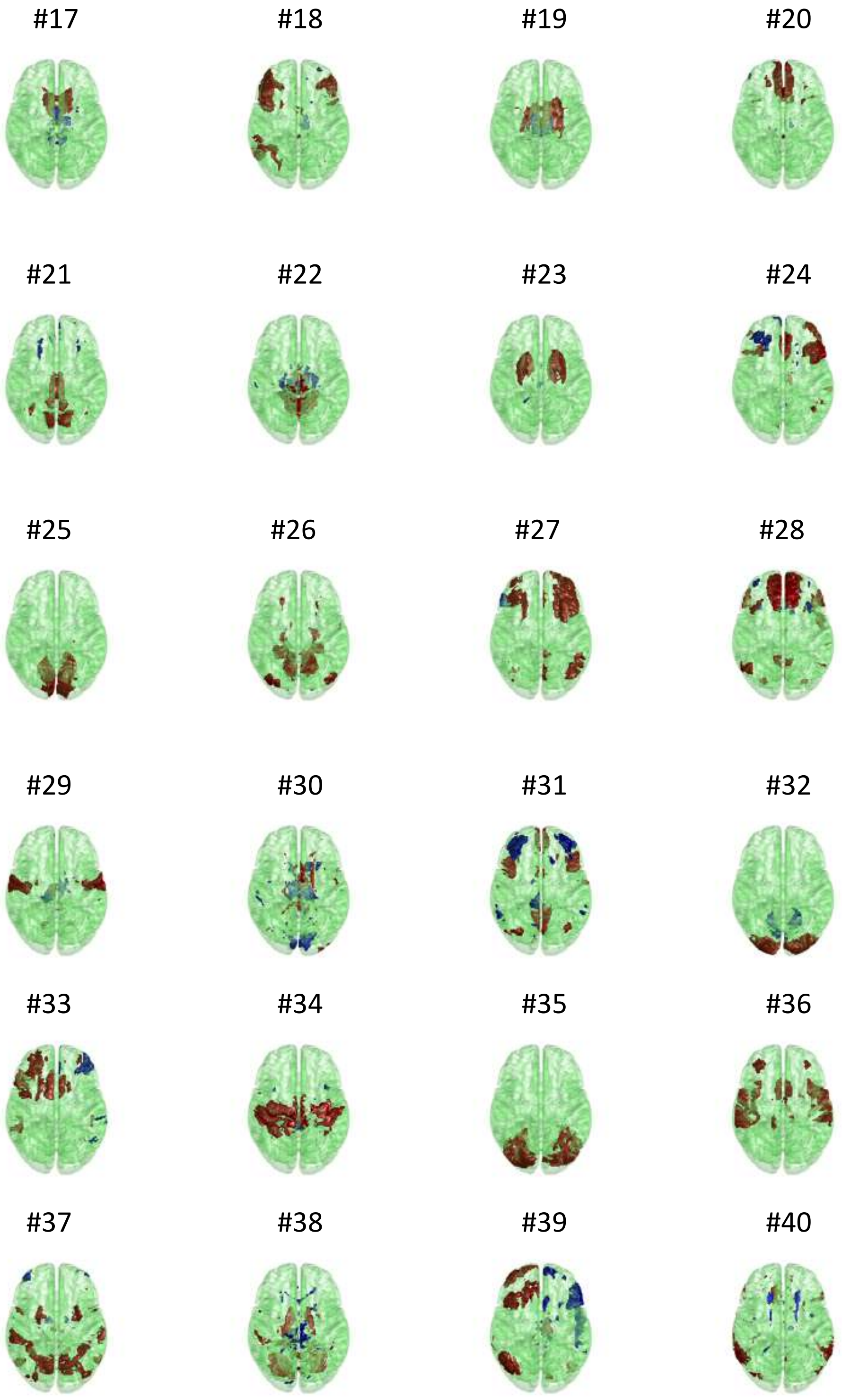
#17
#18
#19
#20
#21
#22
#23
#24
#25
#26
#27
#28
#29
#30
#31
#32
#33
#34
#35
#36
#37
#38
#39
#40

The following are spatial maps of independent components estimated with GIFT when model order is selected as 80.

#1

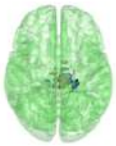

#2

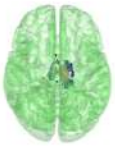

#3

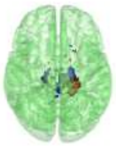

#4

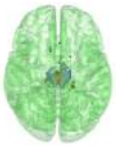

#5

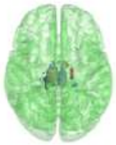

#6

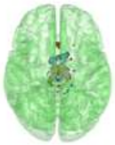

#7

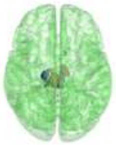

#8

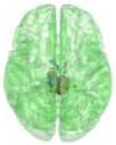

#9

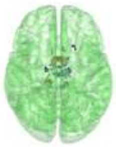

#10

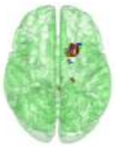

#11

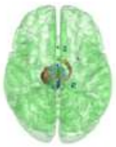

#12

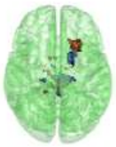

#13

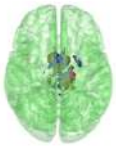

#14

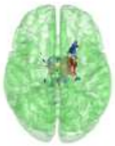

#15

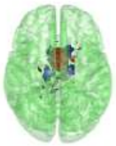

#16

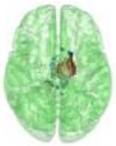

#17

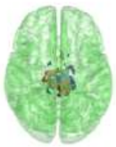

#18

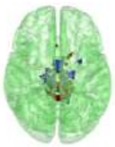

#19

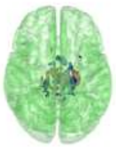

#20

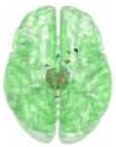

#21

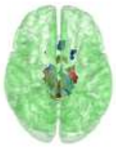

#22

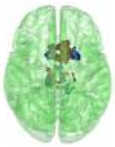

#23

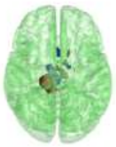

#24

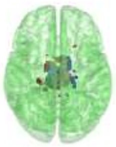

#25

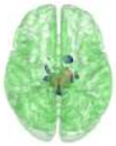

#26

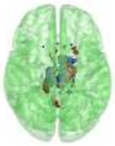

#27

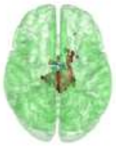

#28

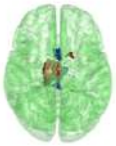

#29

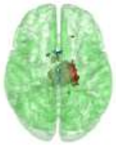

#30

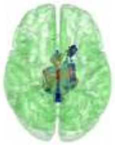

#31

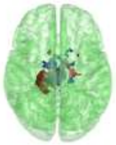

#32

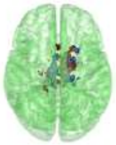

#33

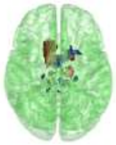

#34

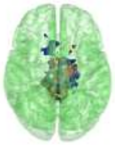

#35

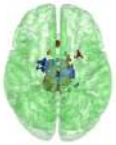

#36

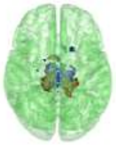

#37

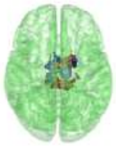

#38

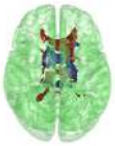

#39

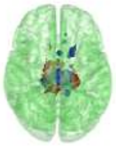

#40

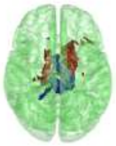

#41

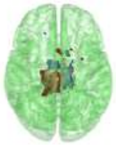

#42

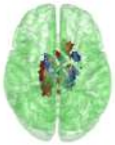

#43

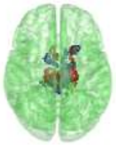

#44

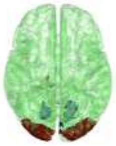

#45

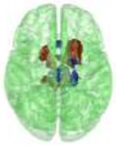

#46

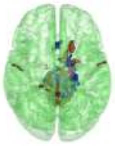

#47

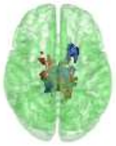

#48

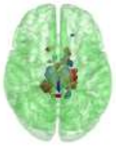

#49

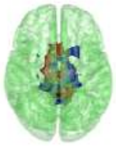

#50

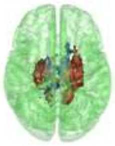

#51

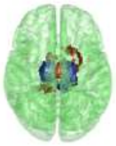

#52

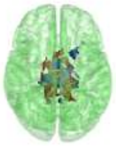

#53

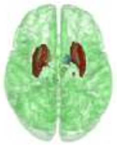

#54

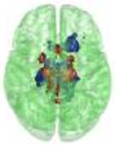

#55

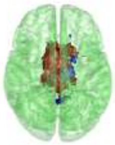

#56

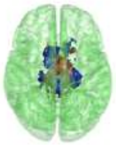

#57

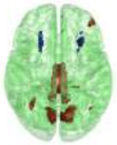

#58

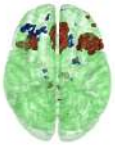

#59

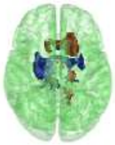

#60

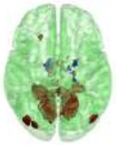

#61

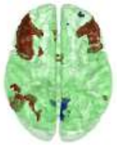

#62

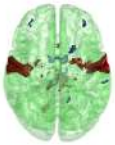

#63

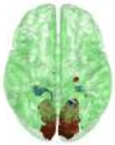

#64

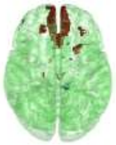

#65

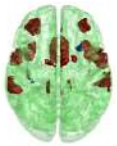

#66

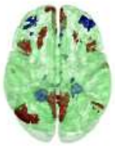

#67

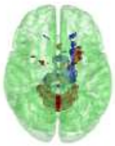

#68

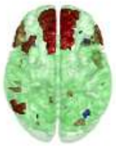

#69

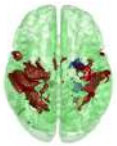

#70

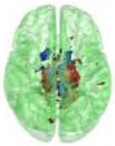

#71

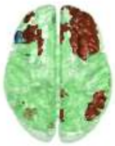

#72

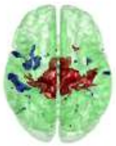

#73

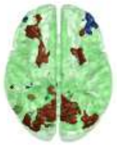

#74

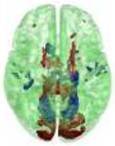

#75

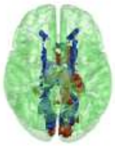

#76

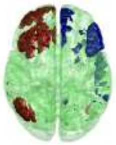

#77

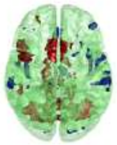

#78

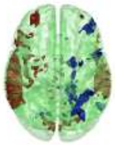

#79

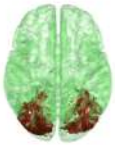

#80

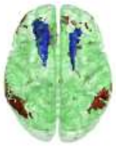

Independent components with MELODIC:

The following are spatial maps of independent components estimated with MELODIC when model order is selected as 40.

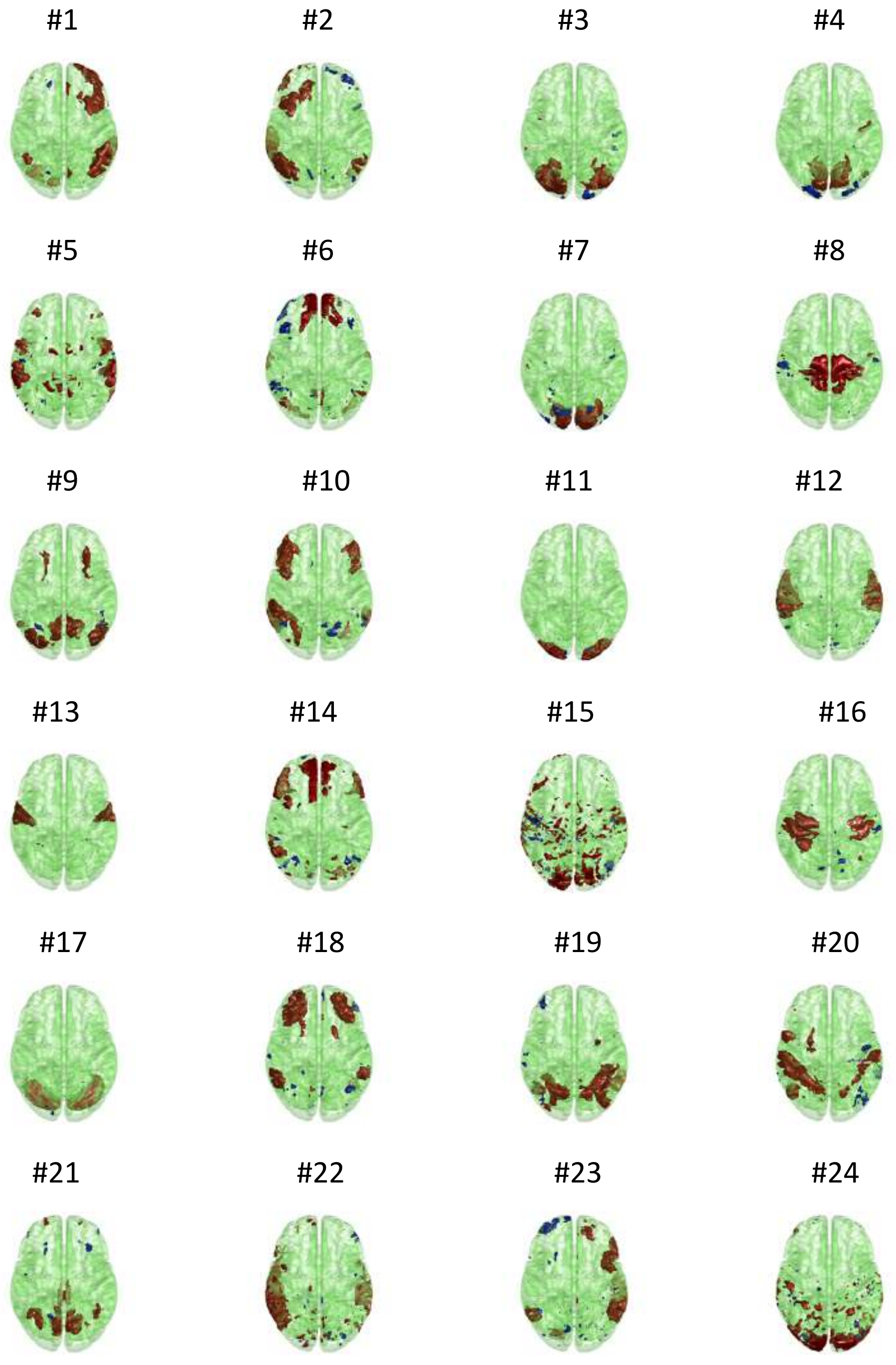

#25 #26 #27 #28

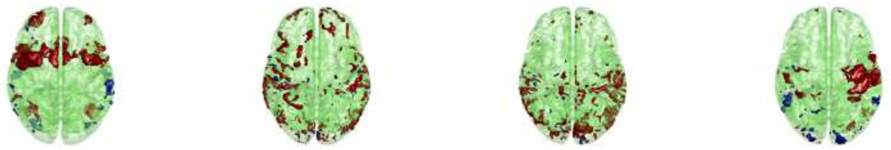

#29 #30 #31 #32

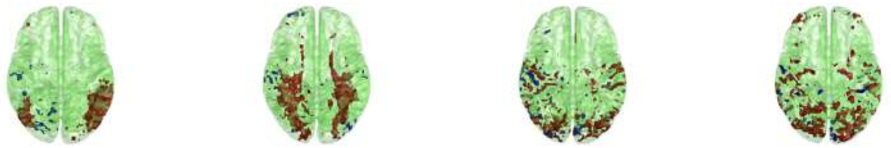

#33 #34 #35 #36

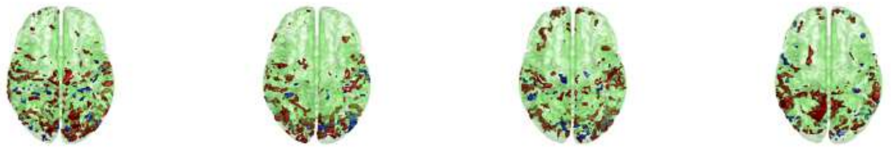

#37 #38 #39 #40

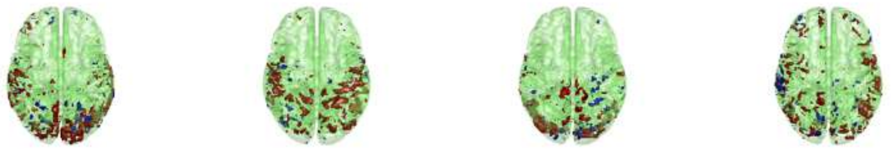

The following are spatial maps of independent components estimated with MELODIC when model order is selected as 80.

#1 #2 #3 #4

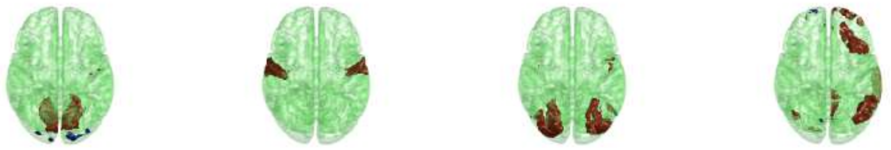

#5 #6 #7 #8

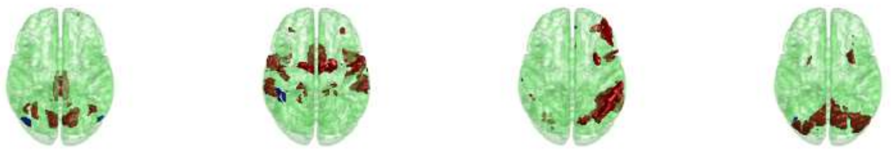

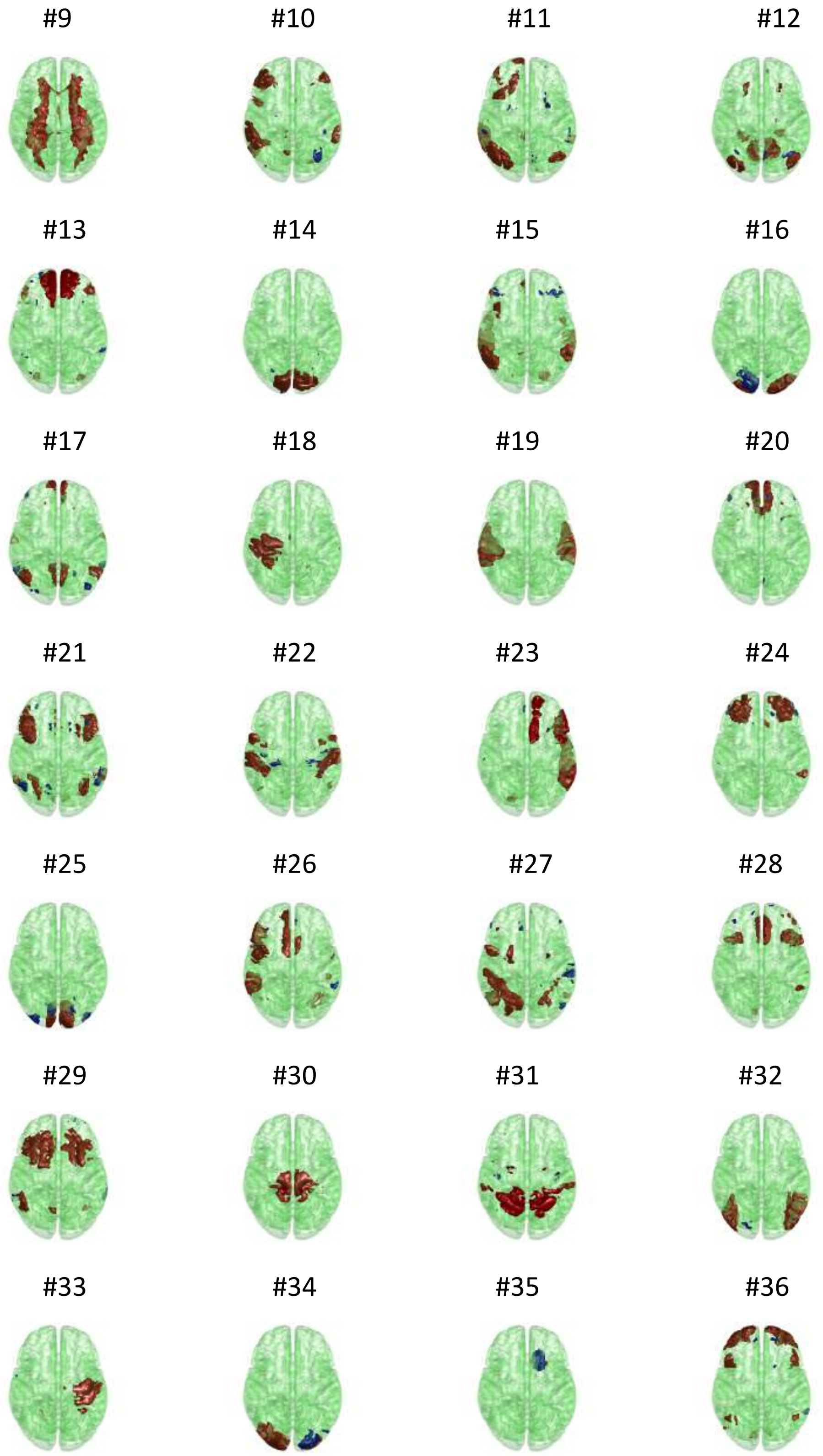
#9
#10
#11
#12
#13
#14
#15
#16
#17
#18
#19
#20
#21
#22
#23
#24
#25
#26
#27
#28
#29
#30
#31
#32
#33
#34
#35
#36

#37

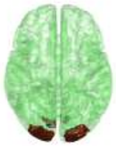

#38

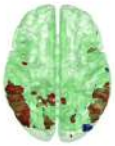

#39

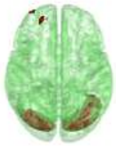

#40

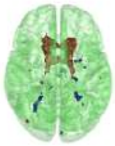

#41

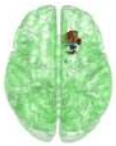

#42

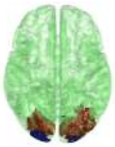

#43

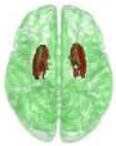

#44

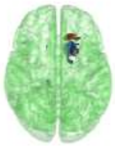

#45

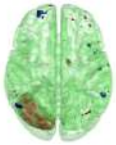

#46

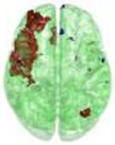

#47

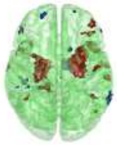

#48

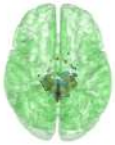

#49

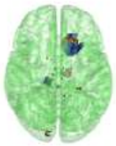

#50

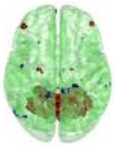

#51

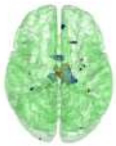

#52

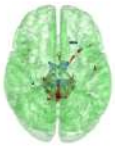

#53

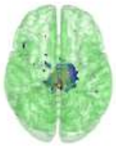

#54

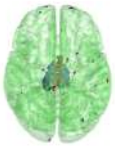

#55

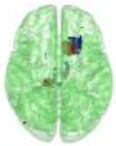

#56

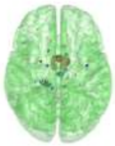

#57

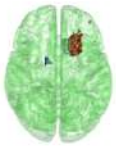

#58

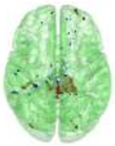

#59

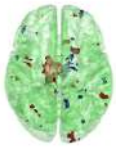

#60

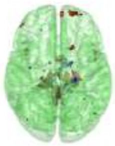

#61

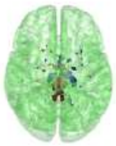

#62

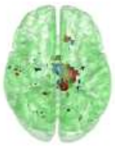

#63

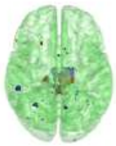

#64

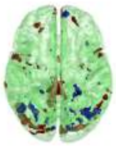

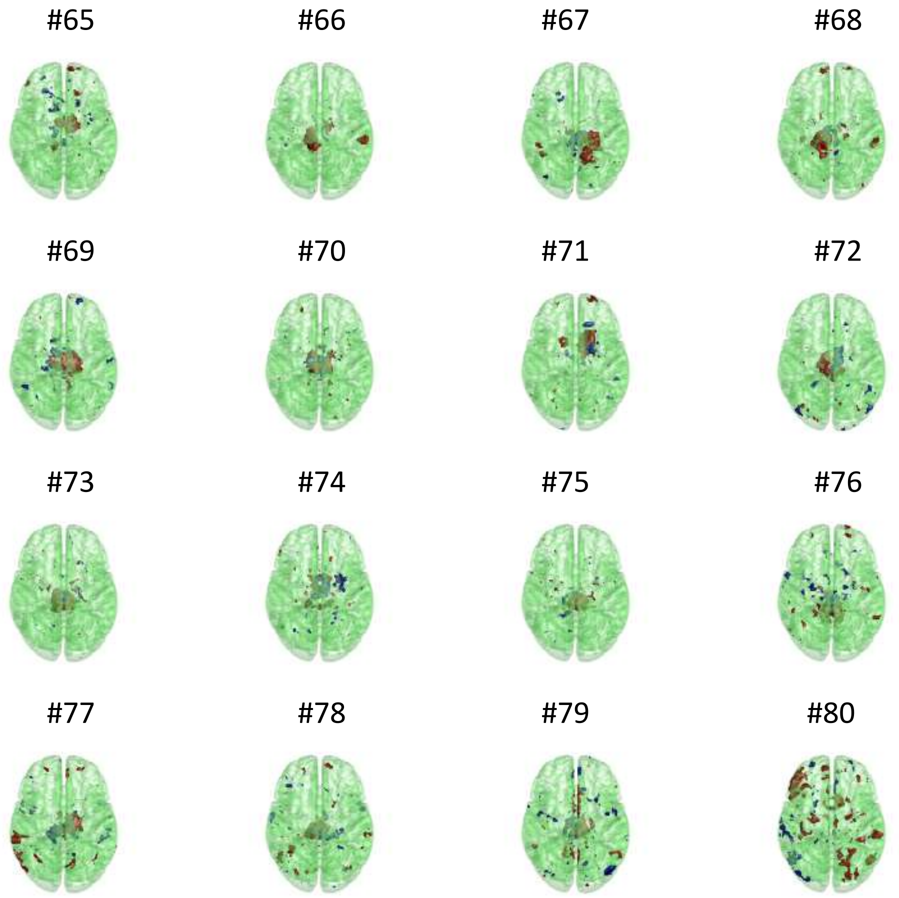
#65
#66
#67
#68
#69
#70
#71
#72
#73
#74
#75
#76
#77
#78
#79
#80

Considering the constraints on conventional spatial ICA that spatial maps are statistically independent, this technique may not easily estimate brain functional networks with spatial overlap (Zhang et al., 2018b). However, in practice, for probabilistic ICA, the spatial sparsity of brain networks combined with background fMRI noise reduces the observed correlation between ICA spatial maps such that even when the brain networks are overlapping, the ICA decomposition can still give a good representation of the signals. Beckmann et al., 2005 discuss this in detail, including a mathematic rationale. Indeed, we frequently observe overlap among spatial maps when applying FSL's probabilistic ICA to group fMRI data, especially for major brain hubs such as medial prefrontal cortex and posterior cingulate. However, to understand how overlap would be handled with noise-free ICA, which is implemented in GIFT and in our Snowball algorithm, we conducted a simulation in which ten resting state network (Smith et al., 2009) ground truth spatial maps were used to generate simulated fMRI data, with two maps having a large area of overlap in the auditory cortex and two maps having a smaller area of overlap in medial prefrontal cortex. We then applied Snowball ICA, MELODIC, and GIFT to assess whether each technique is able to separate the signals accurately under different levels of CNR. Ground truth time series were derived via stage 1 of dual regression (Nickerson et al., 2017), which implements multivariate regression of spatial maps against a single subject's fMRI data to extract time courses for each network. We used this approach to derive ground truth time courses from real fMRI data acquired in 10 health subjects. Overlap between two networks was simulated by altering the spatial maps for two components to include a known overlapping region. Simulated fMRI data for ten subjects was then created according to Nickerson et al (2017), using a range of CNR (0.1-20) as shown in Figure 1. The performance of Snowball ICA, MELODIC and GIFT in network estimation was then assessed by visual inspection, by calculating the spatial cross correlation between ground truth spatial maps and ICA spatial maps, and by calculating Pearson's correlation between timecourses of each network extracted for each participant (via dual regression stage 1; Nickerson et al. 2017) and the ground truth timecourses.

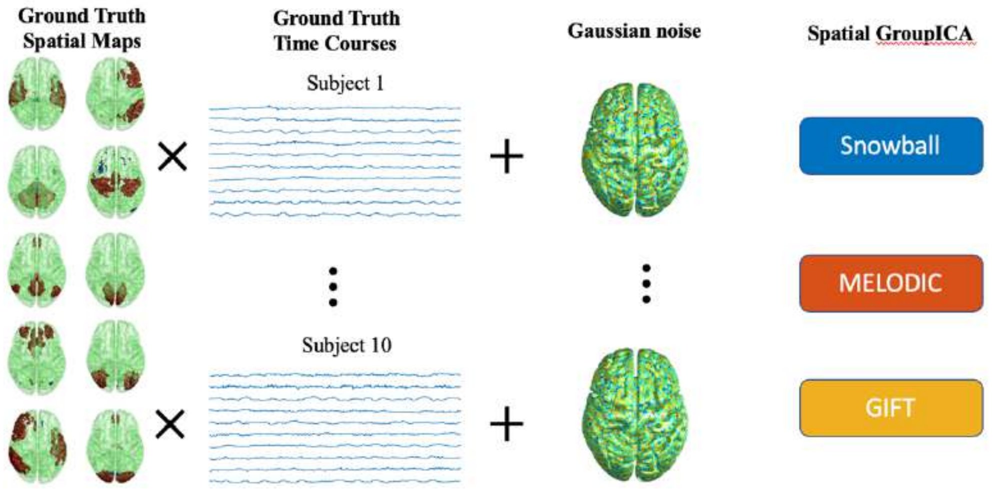

Figure R1 Simulation framework and results for simulated fMRI data with spatial overlap among networks. (a) Ground truth spatial maps were ten well-established resting state networks. Ground truth timecourses were computed vial dual regression stage 1 from real fMRI acquired in 10 healthy subjects. Simulated ground truth fMRI data with varying levels of CNR were then created based on the noisy ICA model and fed into Snowball, MELODIC and GIFT, separately.

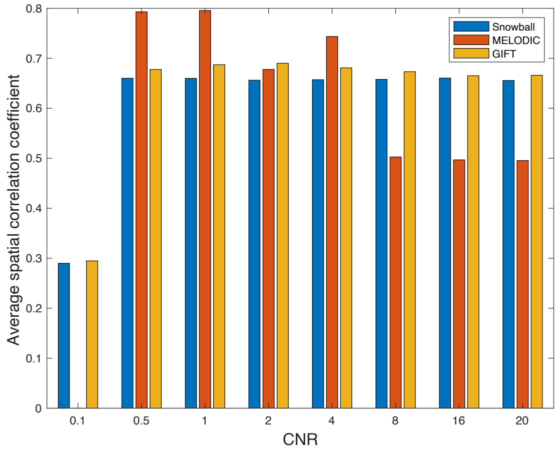

Figure R2 Spatial estimation accuracy of Snowball, MELODIC and GIFT across different levels of contrast-to-noise ratio (CNR).

In the simulated *in vivo* fMRI data experiment, the accuracy of the estimations from Snowball ICA, MELODIC and GIFT are shown in Figure 2. With CNR of 0.1, no components are estimated with MELODIC. The estimations from Snowball and GIFT are also inaccurate. When CNR are 0.5 to 4, MELODIC's probabilistic ICA generally provides more accurate estimates of the components as compared with Snowball and GIFT. When CNR is between 8 and 20, the results from MELODIC are worse than those of MELODIC and GIFT. This is because greater background noise at lower CNRs reduces the observed spatial correlation (Beckmann et al., 2005), resulting in improved ability of probabilistic ICA to estimate components under lower CNR, but worse estimation as the noise levels decrease. Generally, the performance of Snowball ICA is similar to that of GIFT as both algorithms implement noise-free ICA. However, probabilistic ICA could easily be implemented in Snowball ICA. And for typical CNRs, this should give similar results to FSL for separating networks with overlapping regions, although it is noted that Snowball ICA does not require specifying the model order.
