# Supplementary figures and images for: Snowball ICA: A Model Order Free Independent Component Analysis Strategy for Functional Magnetic Resonance Imaging Data
Source: Front Neurosci. 2020 Sep 18;14:569657. doi: 10.3389/fnins.2020.569657 (PMC7530342; doi:10.3389/fnins.2020.569657)

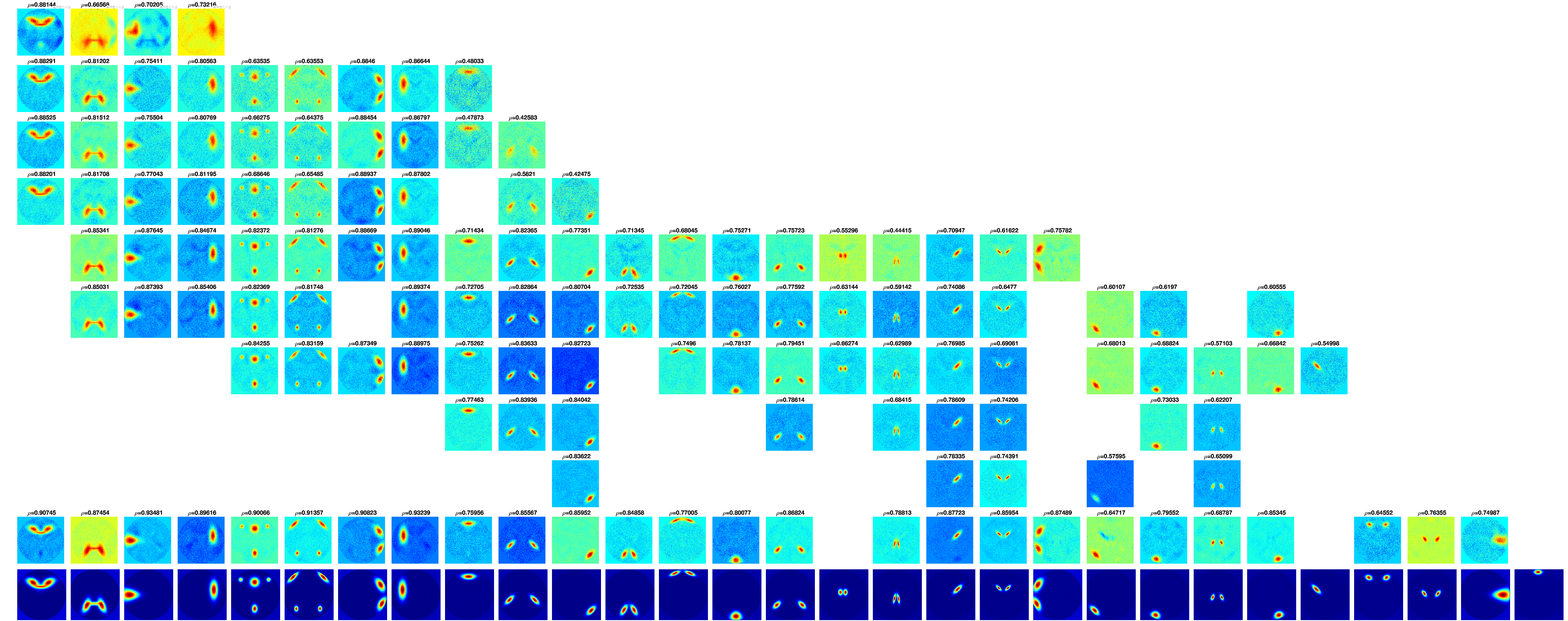

Supplement: Supplementary file 2 [file Image_1.TIFF]
